# Cilia-driven transport in confined ducts: an active porous media model

**Published:** 2026-05-20

**Authors:** JP Raimondi, Feng Ling, Eva Kanso

**Affiliations:** 1 Department of Aerospace and Mechanical Engineering, University of Southern California, Los Angeles, California 90089, USA; 2 School of Physics, Nankai University, Nankai District, Tianjin 300071, China; 3 Department of Physics and Astronomy, University of Southern California, Los Angeles, California 90089, USA

**Keywords:** ciliary flows, active porous media, low-Reynolds-number flows, biological transport, Brinkman equations

## Abstract

Ciliated organs transport viscous fluids through confined ducts, yet how duct morphology and ciliary activity jointly set the limits of flow rate and sustainable pressure remains unclear. Here, we model dense arrays of beating cilia lining duct walls as an active porous medium driven by prescribed metachronal waves, and identify two key morphological parameters that govern transport: the ciliary confinement ratio and the mean ciliary fraction. The resulting flows are described by the incompressible Navier–Stokes–Brinkman equations, which we solve numerically using a spectral method in the low-Reynolds-number regime. We also develop a complementary mean-field analytical model. The active porous medium framework provides an intermediate description between classical envelope theories and filament-resolved simulations and enables a systematic investigation of how fluid transport is shaped by confinement and packing of ciliary material. We find that transport is characterized by a decreasing linear relationship between flow rate and pressure generation, marking a fundamental trade-off between throughput and sustainable adverse pressure. These results provide a unified physical interpretation of the morphological diversity of ciliated ducts, from high-throughput ciliary carpets to pressure-generating ciliary flames, and offer guiding principles for the design of bio-inspired microfluidic pumps.

## 1. Introduction

Ciliated organs drive essential transport processes throughout animal physiology. In humans, coordinated ciliary beating directs cerebrospinal fluid in the brain ventricles (Faubel et al. 2016), transports gametes in the reproductive tract (Greenstone & Cole 1985; Satir & Christensen 2007), and clears mucus in the airways (Afzelius 1976; Roth et al. 2025). These functions arise from the collective activity of dense arrays of motile cilia. Motile cilia are micron-scale active filaments whose oscillatory motion generates fluid flow in the viscous regime at vanishingly small Reynolds numbers (Lauga & Powers 2009; Gilpin et al. 2020). Despite decades of work on ciliary beat kinematics and synchronization, a fundamental question remains open: how do ciliary activity and duct morphology jointly set the limits of fluid transport, in terms of achievable flow rate and pressure generation? Recent evidence points to a tight coupling between ciliated duct morphology and transport function across diverse biological systems (Ling et al. 2024), yet a predictive quantitative framework linking beat kinematics, metachronal wave coordination, and duct geometry to fluid transport remains incomplete.

**Figure 1.**
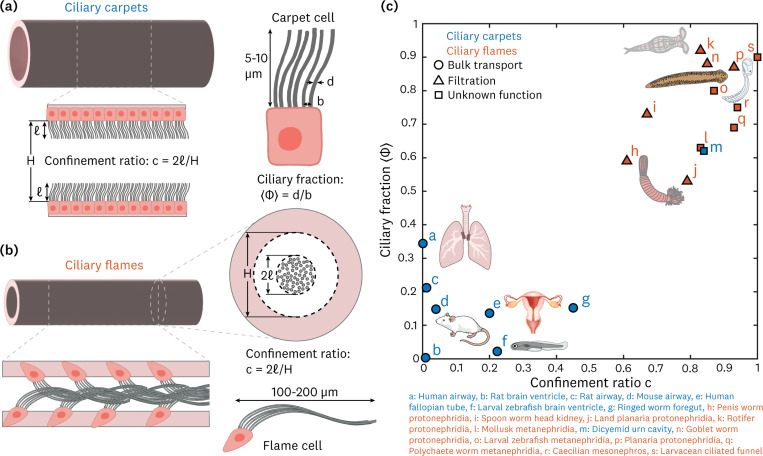
Ciliated systems, corresponding geometric measures, and biological data. The ciliary confinement ratio, c, denotes the ratio of the ciliary layer thickness to the lumen diameter. The ciliary fraction, ⟨Φ⟩, defines the corresponding fraction of cilia within the ciliated region. (a) **Ciliary carpets** tend to have large lumen (50–1000, *μ*m) with short (5–10, *μ*m), wall-normal cilia. Activity is distributed over the boundary and quantified from cross-sections parallel to the lumen axis. (b) **Ciliary flames** have small lumen (1–50, *μ*m) with long (100–200, *μ*m), axially aligned cilia. Ciliary activity is concentrated near the center of the lumen and quantified from cross-sections perpendicular to the axis. (c) Measurements of c and ⟨Φ⟩ from biological data, showing distinct regimes: carpets occupy low c and ⟨Φ⟩, while flames exhibit high values of both. Marker shape indicates function: circles for bulk transport, triangles for filtration, and squares for ducts with unknown functions. See table 4 for full dataset and references. Lung and rat illustrations from NIAID NIH BioArt Source (bioart.niaid.nih.gov/bioart/231) and (bioart.niaid.nih.gov/bioart/54), female reproductive tract © blueringmedia / Adobe Stock, rotifer © Kazakova Maryia / Adobe Stock, others are original.

In vertebrate organs, short cilia form dense carpets that beat predominantly normal to the epithelial surface, generating metachronal waves well suited for high-throughput transport and mixing in wide lumens (Ding et al. 2014; Nawroth et al. 2017; Gilpin et al. 2020; Kanale et al. 2022) (figure 1a). In contrast, some invertebrates exhibit ciliary flames, in which long, tightly packed cilia beat longitudinally within narrow ducts, pumping fluid through highly resistive pathways in filtration and excretion organs (Vogel 2007; Ling et al. 2024) (figure 1b). Although these configurations differ markedly in geometry and function, both operate in the viscous regime and rely on collective traveling-wave kinematics to drive transport. Comparative surveys across animal phyla indicate that ciliary carpets and ciliary flames represent two extremes in ciliary architecture (Ling et al. 2024): carpets are adapted for high-throughput transport and mixing, whereas flames are adapted for filtration and sustained pumping against adverse pressure gradients. Together, these systems illustrate how duct geometry and ciliary organization map onto distinct transport tasks, motivating the need for a unified physical description capable of spanning this diversity.

To place these architectures on a quantitative footing, we compile geometric data from published measurements, drawing in particular on Ling et al. (2024). We characterize each system using two dimensionless parameters: the ciliary confinement ratio c, defined as the ratio of the ciliary layer height to the total duct height, and the mean ciliary fraction ⟨Φ⟩, defined as the fraction of the ciliary layer occupied by ciliary material. The compiled data (figure 1c, table 4, and appendix A) show that ciliary carpets occupy a regime of low confinement and moderate ciliary fraction, whereas ciliary flames lie at high confinement and high ciliary fraction.

These morphological trends raise a natural question: given a ciliated channel characterized by confinement ratio c and mean ciliary fraction ⟨Φ⟩, how do these geometric parameters, together with prescribed ciliary kinematics, determine the resulting flow rate and the maximum pressure that can be sustained?

Classical theoretical approaches to cilia-driven flows trace back to Taylor’s swimming sheet (Taylor 1951) and Blake’s envelope model (Blake 1971, 1972), in which densely packed ciliary carpets are represented as an impermeable surface supporting traveling waves. These models, and their extensions (Michelin & Lauga 2011; Smith et al. 2008; Chrispell et al. 2013; Ramirez-San Juan et al. 2020; Liu et al. 2025a,b,c, 2026a), have provided fundamental insight into propulsion and transport, but neglect flow penetration through the ciliary layer and its internal structure. At the opposite extreme, filament resolved models capture individual cilium mechanics and hydrodynamic interactions (Liron & Mochon 1976; Liron 1978; Gueron & Liron 1992; Blake et al. 1982; Ding et al. 2014; Guo et al. 2014; Nawroth et al. 2017; Guo et al. 2020; Osterman & Vilfan 2011), but become computationally prohibitive and parameter-rich when applied to realistic systems.

A promising intermediate description models dense ciliary assemblies as an active porous medium governed by a Brinkman-type equation (Stein & Shelley 2019; Wuttanachamsri & Schreyer 2020, 2021; Ling et al. 2024; Dutta et al. 2024). In this framework, the collective effect of many beating filaments is coarse-grained into a distributed body force and an effective drag.

Here, we build on this approach to develop a unified Navier–Stokes–Brinkman model for ciliary pumping in confined channels. The channel is represented as a multi-layer system, with ciliary regions modeled as active porous layers endowed with prescribed traveling-wave kinematics. This formulation occupies an intermediate level between envelope models and filament-resolved simulations, retaining permeability and distributed forcing while avoiding explicit resolution of individual cilia. It enables a systematic investigation of how cilia length, packing density, metachronal coordination, and channel geometry jointly determine transport across the full spectrum from carpets to flames.

## 2. Problem formulation

**Figure 2.**
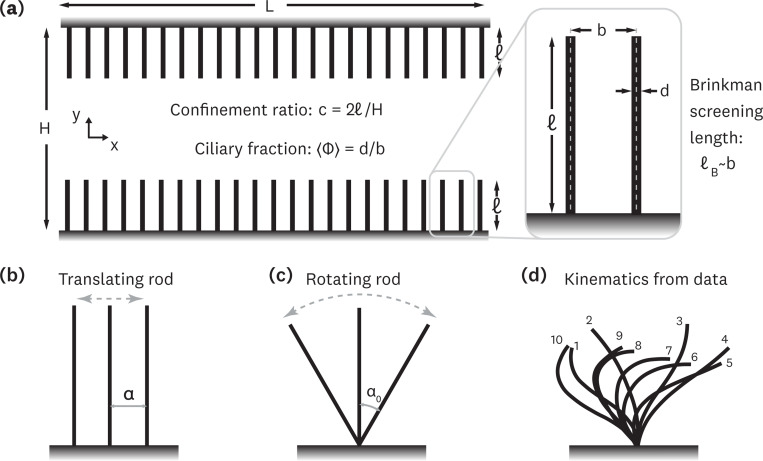
Reference configuration and ciliary kinematics. (a) Undeformed reference configuration of the active Brinkman channel, with channel length L, height H, and ciliary layer thickness ℓ. The ciliary confinement ratio is defined as c=2ℓ/H. Inset: cilia of diameter d are uniformly spaced with center-to-center distance b, giving an average ciliary fraction ⟨Φ⟩=d/b. The Brinkman screening length $\ell_{B}$ is proportional to b. (b) Translating-rod kinematic model with oscillation amplitude a. (c) Rotating-rod kinematic model with oscillation amplitude $\alpha_{0}$. (d) Experimentally measured ciliary kinematics in rabbit trachea (Sanderson & Sleigh 1981); snapshots of the motion are evenly spaced over one beat cycle.

We consider cilia-driven flow in a rectangular channel of length L and height H, with ciliary carpets lining the side walls (figure 2). Lengths are nondimensionalized by L and time by L/U, where U=ωℓ is a characteristic ciliary speed defined by the beat frequency ω and ciliary layer thickness ℓ.

### 2.1. Fluid model

We adopt a coarse-grained continuum description in which the coupled ciliary activity and fluid flow are modeled as an active porous medium (Stein & Shelley 2019). The dynamics are governed by the incompressible Navier–Stokes equations with an active Brinkman drag term, given by the nondimensional form

(2.1) $$Re\left(\frac{\partial u}{\partial t}+u\cdot \nabla u\right)=-\nabla p+\nabla^{2}u-B\left(u-v_{c}\right)-\Delta P\;e_{x},\;\nabla \cdot u=0.$$

Here, t denotes time, and x and y are the streamwise and transverse coordinates, respectively, with $e_{x}$ the unit vector in the streamwise direction. The fluid velocity and pressure fields are denoted by u(x,y,t) and p(x,y,t), with the latter enforcing incompressibility.

The Reynolds number is defined as Re=ρUL/μ, where ρ and μ are the fluid density and viscosity. A uniform pressure gradient ΔP is imposed opposite to the desired direction of transport.

In (2.1), the term $B\left(u-v_{c}\right)$ represents an effective momentum exchange between the fluid and the actively driven porous medium. The contribution -Bu corresponds to viscous drag passively induced by the porous medium, while $Bv_{c}$ acts as an internal forcing that can drive flow even in the absence of external pressure gradients. Together, these terms act to force the fluid velocity u to match the imposed porous medium velocity $v_{c}$ with the strength of this forcing being set by the Brinkman coefficient, B. Both $v_{c}(x,y,t)$ and B(x,y,t) vary in space and time as prescribed by the ciliary motion (section 2.2) and are nonzero only within the ciliary layers, vanishing elsewhere.

The field $v_{c}$ encodes the ciliary kinematics, including individual beating and metachronal wave propagation. These motions deform the porous medium and thereby modulate the local ciliary fraction Φ(x,y,t), defined as the fraction of the ciliated layer occupied by ciliary material. The Brinkman coefficient B depends on Φ and quantifies viscous drag.

In the Stokes–Brinkman limit (Re = 0), this drag replaces the long-ranged Stokeslet Green’s function of the Stokes equations, which decays as 1/r, with a screened Green's function that decays as $e^{-r/\ell_{B}}/r$. The parameter $\ell_{B}$ is the Brinkman hydrodynamic screening length, which sets the distance over which flow disturbances propagate in the effective porous medium. In the present model, $\ell_{B}={\left\langle B\right\rangle }^{-1/2}$, where ⟨B⟩ denotes the spatial average of the Brinkman coefficient.

To close the model, we prescribe planar ciliary motion (section 2.2) and consider periodic boundary conditions in the longitudinal x-direction, no-slip boundary conditions in the transverse y-direction, and assume that the flow is invariant in the out of plane z-direction, such that

(2.2) u(0,y,t)=u(L,y,t),u(x,0,t)=u(x,H,t)=0,p(0,y,t)=p(L,y,t).

Cilia-driven flows typically occur at low Reynolds numbers, Re = 10^−4^–10^−1^, where viscous forces dominate and inertial effects are weak (Ding et al. 2014; Nawroth et al. 2017; Gilpin et al. 2020; Kanale et al. 2022). However, unsteady beating and strong confinement can generate finite advective and inertial contributions (Wei et al. 2019, 2021).

From a numerical standpoint, solving the time-dependent Navier–Stokes equations at small but finite Reynolds number provides a stable and well-conditioned framework for capturing unsteady low-Re flows. We therefore retain the full Navier–Stokes–Brinkman system (2.1) and fix Re = 0.01, which remains in the viscous-dominated regime while allowing for weak inertial effects. Here, we use an implicit-explicit spectral method to solve (2.1,2.2). Details of the numerical method, together with convergence tests and validation, are provided in appendices B and C, algorithms B.1 and B.2, and figures 12–16.

### 2.2. Ciliary kinematics

We next outline a procedure for constructing the continuum fields in (2.1): the Brinkman coefficient B(x,y,t) and the ciliary velocity $v_{c}(x,y,t)$, both determined by the geometry and kinematics of the ciliary system.

It is convenient to introduce an undeformed, uniform reference configuration consisting of a discrete ciliary carpet extending over the channel length L, with ciliary layer thickness ℓ, diameter d, and intercilium spacing b. In this uniform state, the ciliary fraction is

(2.3) $$\langle \Phi \rangle =\frac{L}{b}\frac{A_{c}}{A}=\frac{d}{b},$$

where L/b is the number of cilia, $A_{c}=\ell d$ is the area of a single cilium, and A=ℓL is the total carpet area.

To set the scale of the mean Brinkman drag coefficient ⟨B⟩, we estimate the drag force coefficient in the ciliary layer as follows. Using slender-body theory, we approximate the drag force $F_{D}=c_{D}U$ on a single cilium in a uniform flow of speed U directed perpendicular to its axis. The drag coefficient is $c_{D}=4\pi \mu \ell /ln(2\ell /d)$ (with μ=1 in the nondimensional formulation). Neglecting hydrodynamic interactions with the anchoring wall and between neighboring cilia, the total drag exerted by the ciliary layer scales linearly with the number of cilia. The corresponding drag coefficient is therefore $C_{D}=c_{D}L/b=4\pi \mu \ell L/b\;ln(2\ell /d)$.

The drag coefficient $C_{D}$ converts velocity to force while the Brinkman coefficient B converts velocity to pressure gradient. Thus, to obtain the Brinkman coefficient ⟨B⟩, we divide $C_{D}$ by an effective volume. Given that drag acts on the pore scale (b-d) and is distributed over the carpet area ℓL, we divide $C_{D}$ by ℓL(b-d). Substituting (2.3) into the expression for $C_{D}$, we arrive at the Brinkman drag coefficient in the undeformed reference configuration

(2.4) $$\langle B\rangle =\frac{4\pi \mu }{d(b-d)\;ln(2\ell /d)}\langle \Phi \rangle .$$

Ciliary motion deforms the reference configuration, giving rise to spatially and temporally varying fields B(x,y,t) and $v_{c}(x,y,t)$ that reflect both the geometry and kinematics of the ciliary carpet. We therefore seek expressions relating these Eulerian fields to the reference Brinkman coefficient ⟨B⟩ and the prescribed ciliary motion.

Let (X,Y) denote Lagrangian material coordinates, and let the reference and current (deformed) configurations of the cilia be related by a forward kinematic mapping $\chi_{c}(X,Y,t)$ that maps each material point (X,Y) to its Eulerian position (x,y) at time t. Conversely, the inverse mapping $\chi_{c}^{-1}(x,y,t)$ assigns each fixed Eulerian point (x,y) to the corresponding Lagrangian label (X,Y) of the cilium occupying that location at time t

(2.5) $$\chi_{c}(X,Y,t)=\left[\begin{array}{l} x(X,Y,t)\\ y(X,Y,t) \end{array}\right],\;\chi_{c}^{-1}(x,y,t)=\left[\begin{array}{l} X(x,y,t)\\ Y(x,y,t) \end{array}\right].$$

The ciliary velocity field in the Lagrangian frame is given by

(2.6) $$v_{c}\left(X,Y,t\right)=\frac{\partial \chi_{c}\left(X,Y,t\right)}{\partial t}.$$

The deformation gradient is $F=\nabla_{X}\chi_{c}$ with determinant J=det(F), which measures the local area change induced by the ciliary deformation. This allows the ciliary fraction ⟨Φ⟩ and Brinkman coefficient to be mapped from the reference to the deformed configuration, yielding

(2.7) $$B\left(X,Y,t\right)=\frac{\left\langle B\right\rangle }{J\left(X,Y,t\right)}.$$

In regions where the active porous medium is locally compressed (J<1), the Brinkman drag B is enhanced relative to the reference state, whereas in stretched regions (J>1) it is reduced.

In (2.6) and (2.7), the fields $v_{c}$ and B are expressed in terms of Lagrangian coordinates (X,Y), while the Navier-Stokes-Brinkman equations (2.1) and boundary conditions (2.2) are formulated in Eulerian coordinates (x,y). Using the inverse mapping $\chi_{c}^{-1}(x,y,t)$, we obtain

(2.8) $$v_{c}(x,y,t)={\left.\frac{\partial \chi_{c}}{\partial t}\right|}_{\left(X(x,y,t),\;Y(x,y,t)\right)},\;B(x,y,t)={\left.\frac{\left\langle B\right\rangle }{J}\right|}_{\left(X(x,y,t),\;Y(x,y,t)\right)}.$$

In summary, this procedure defines a uniform reference configuration and a kinematic mapping $\chi_{c}$ that together yield continuous fields B(x,y,t) and $v_{c}(x,y,t)$ capturing the spatiotemporal variations induced by ciliary motion. The framework is general and can be extended to three-dimensional settings and heterogeneous reference configurations. We apply it in section 3 to idealized kinematic models and experimentally reconstructed ciliary motion.

Lastly, for later use, we introduce a measure of the total ciliary material in the channel defined by the spatial integral of the local ciliary fraction Φ(x,y,t). Conservation of material implies that the total ciliary content is invariant under deformation. Accordingly, the integral of Φ over the current configuration is equal to that of the reference configuration, yielding a total ciliary material proportional to 2ℓL⟨Φ⟩. For fixed cilia length ℓ and channel length L, the reference ciliary fraction ⟨Φ⟩ therefore uniquely determines the total amount of ciliary material in the system.

## 3. Numerical simulations

To demonstrate the versatility of our modeling framework, we consider three planar models of ciliary motion with increasing kinematic complexity (figure 2b-d and table 3). In all cases, individual cilia undergo periodic oscillations at a frequency ω with a systematic phase lag along the longitudinal $e_{x}$-direction of the carpet, giving rise to a traveling metachronal wave. The metachronal wavenumber k sets the phase lag between neighboring cilia and thus the wavelength of the traveling wave. Throughout, we take the wavenumber k=2π/L, so that one metachronal wavelength spans the channel length L, with positive k corresponding to waves propagating from right (positive x) to left (negative x). Finally, we restrict the kinematic parameters such that the mapping χ remains locally invertible in all cases and the active porous medium does not fold over itself; that is, we constrain the Jacobian to be strictly positive J>0.

The simplest model (figure 2b) represents each cilium as a rigid vertical rod undergoing symmetric horizontal oscillations, with a spatially varying phase θ(X,t) corresponding to a metachronal wave of wavenumber k in the longitudinal direction,

(3.1) $$\chi_{c}(X,Y,t)=\left[\begin{array}{c} X+a\;cos\;\theta \\ Y \end{array}\right],\;\theta (X,t)=\omega t+kX.$$

**Figure 3.**
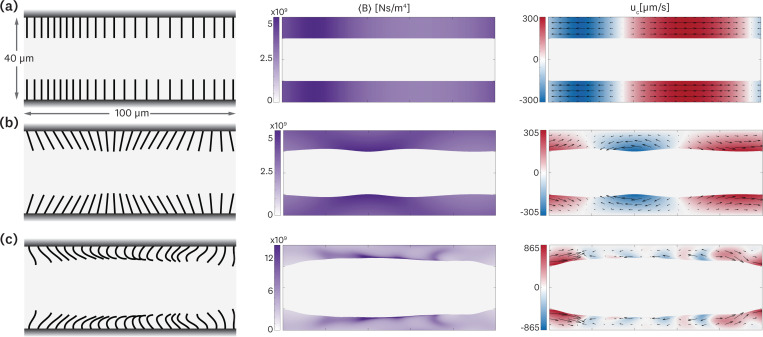
Discrete ciliary carpets and corresponding continuum fields. Left to right: time snapshots of the discrete rod representation (number of rods not to scale), Brinkman coefficient field, and ciliary velocity field (colormap showing streamwise component $u_{c}=v_{c}\cdot e_{x}$). (a) Translating rod model, with a=5μm. (b) Rotating rod model, with $\alpha_{0}=\pi /6$. (c) Kinematics reconstructed from experimental measurements (Sanderson & Sleigh 1981). In all cases, a single metachronal wave θ(X,t)=ωt+kX with k=2π/L is imposed over the channel length. Parameter values: =10Hz, b=1μm, d=0.2μm, ℓ=10μm, L=100μm, H=40μm, ⟨Φ⟩=0.2, and $\langle B\rangle =3.4\times 10^{5}$.

Here, X labels the position along the carpet and thus indexes individual cilia, while Y denotes arclength along each cilium measured from its base. The parameters a and ω are the non-dimensional oscillation amplitude and frequency, respectively. The condition J>0 reduces to ak<1. Because the mapping (3.1) is transcendental in X, its inverse cannot be obtained in closed form. We therefore compute $\chi_{c}^{-1}$ numerically using a pointwise one-dimensional Newton iteration and evaluate the Eulerian fields $v_{c}(x,y,t)$ and B(x,y,t) (algorithm B.1). Figure 3a depicts snapshots of the Brinkman drag field (center column) and corresponding ciliary velocity field (right column). Both fields translate smoothly and periodically in the direction of metachronal wave propagation, from right to left. The Brinkman field captures the local stretching and compression of the carpet: compressed regions correspond to cilia in their backward stroke, whereas stretched regions correspond to cilia in their forward stroke.

The second kinematic model describes a rigid cilium undergoing symmetric angular oscillations about its base (figure 2c), also with a spatially varying phase θ(X,t)=ωt+kX,

(3.2) $$\chi_{c}\left(X,Y,t\right)=\left[\begin{array}{c} X+Y\;\sin\;\alpha \\ Y\;\cos\;\alpha \end{array}\right],\;\alpha =\alpha_{o}\sin\;\theta .$$

Here, α(X,t) is the instantaneous tilt angle, $\alpha_{o}$ is the angular amplitude, and ω and k again set the beat frequency and metachronal wavelength. This motion results in carpet deformations in both the x and y directions (figure 3b), with no-slip boundary conditions at the channel walls.

The third and most realistic model (figure 2d) is based on experimentally measured planar ciliary waveforms, with asymmetric power and recovery strokes, and again with θ(X,t)=ωt+kX,

(3.3) $$\chi_{c}\left(X,Y,t\right)=\left[\begin{array}{c} X+x_{c}\left(Y,\theta \right)\\ y_{c}\left(Y,\theta \right) \end{array}\right].$$

Here, the functions $x_{c}(Y,\theta )$ and $y_{c}(Y,\theta )$ are represented by polynomial expansions in arclength and Fourier series in phase θ(X,t), with coefficients $A_{0x}$, $A_{0y}$, $A_{x}$, $A_{y}$, $B_{x}$, $B_{y}$ optimized to fit experimental measurements of rabbit trachea ciliated epithelium (Fulford & Blake 1986; Sanderson & Sleigh 1981),

(3.4) $$\begin{array}{l} x_{c}(Y,\theta )=\ell \sum_{m=1}^{M}{\left(\frac{Y}{\ell }\right)}^{m}\left[\frac{1}{2}A_{0x}^{\left(m\right)}+\sum_{n=1}^{N}\left(A_{x}^{\left(m,n\right)}\;cos(n\theta )+B_{x}^{\left(m,n\right)}\;sin(n\theta )\right)\right],\\ y_{c}(Y,\theta )=\ell \sum_{m=1}^{M}{\left(\frac{Y}{\ell }\right)}^{m}\left[\frac{1}{2}A_{0y}^{\left(m\right)}+\sum_{n=1}^{N}\left(A_{y}^{\left(m,n\right)}\;cos(n\theta )+B_{y}^{\left(m,n\right)}\;sin(n\theta )\right)\right]. \end{array}$$

This representation captures the full time-dependent shape of each cilium and the propagation of metachronal waves along the carpet (figure 3c). For the same reference ciliary fraction and metachronal wavelength, this ciliary kinematics yields a maximum Brinkman coefficient more than twice as large as that of the translating and rotating rod cases because it results in large ciliary fractions near the surface of the carpet. It also produces larger peak ciliary velocities, even with identical beat frequency and comparable oscillation amplitudes, owing to its distinct fast power strokes and slow recovery strokes.

With the inputs to the Navier-Stokes-Brinkman equation (2.1), B(x,y,t) and $v_{c}(x,y,t)$, in hand, we solve (2.1) subject to boundary conditions (2.2) using an implicit-explicit spectral method that represents u with Fourier modes in the x direction and sine modes in the y direction, automatically satisfying (2.2). The full details of our numerical method and validation are provided in appendices B-C. Owing to the traveling wave form of the inputs, the flow field is also a traveling wave. Thus for each simulation, we advance the solution forward in time until a periodic steady state is reached.

**Figure 4.**
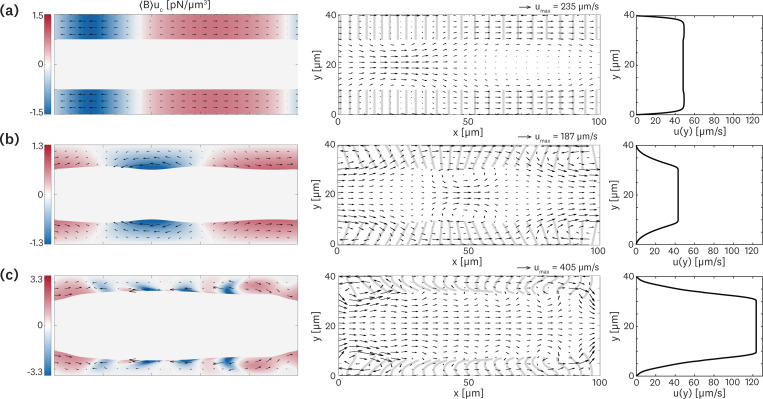
Flows driven by prescribed ciliary activity. Left: instantaneous active porous media force density, $Bv_{c}$, colormap indicates streamwise component $Bv_{c}\cdot e_{x}$ in units of $pN/\mu m^{3}$. Middle: instantaneous velocity fields at zero applied pressure, scaled by the maximum speed (indicated). Grey rods denote the extent of the ciliary carpet (rod spacing not to scale). Right: streamwise velocity profiles averaged over one beat cycle. Corresponding ciliary activity depicted in figure 3 and table 3. (a) Translating rod model resulting in pumping rate $Q=1867\;\mu m^{2}s^{-1}$. (b)Rotating rod model, $Q=1228\;\mu m^{2}s^{-1}$. (c) Experimentally reconstructed kinematics, $Q=3540\;\mu m^{2}s^{-1}$. ΔP=0, all other parameter values are the same as those in figure 3.

In figure 4, we show snapshots of the active force density $Bv_{c}$ (left column) for the three ciliary kinematic models in figure 3, each with identical reference configurations and channel geometries. Given that a single respiratory cilium generates approximately 60 pN of force at its tip (Hill et al. 2010), the active force densities generated by our model are of the correct order of magnitude for ciliary carpets. Although the prescribed motions in the translating (3.1) and rotating (3.2) rod models are symmetric with respect to the Lagrangian label X at the level of individual cilia, the traveling metachronal waves arising from the phase lag between neighboring cilia break translational symmetry in the Eulerian frame. As a result, the force densities are biased, with a larger portion of the forcing being directed in the positive x direction.

The numerically obtained steady state flowfields at zero applied pressure (figure 4 middle column) reflect this bias, with each model driving a net fluid flux. To quantify this flux, we compute the dimensional mean streamwise velocity profile u(y) and corresponding volumetric flow rate Q

(3.5) $$u\left(y\right)=\frac{1}{L}\int_{0}^{L}u\left(x,y,t\right)\cdot e_{x},dx,\;Q=\int_{0}^{H}u\left(y\right),dy,$$

with Q expressed per unit depth. Streamwise flow profiles for model are plotted in figure 4(right column). Because the steady-state velocity field is both spatially and temporally periodic, time-averaging over one beat cycle is equivalent to spatial averaging over one metachronal wavelength (appendix B and figure 12a,b).

Despite sharing the same reference ciliary fraction and metachronal wavelength, as well as identical beat frequency and comparable oscillation amplitudes, the experimentally measured kinematics generate a substantially larger maximal force density, flow velocity, and flow rate compared to the symmetrically oscillating rod models. Importantly, the three cases also exhibit distinct flow topologies. Purely translational oscillations produce little to no recirculation within the channel, whereas rigid rotational oscillations generate recirculation zones in the channel core that are detrimental to net transport. In contrast, the experimentally measured kinematics induce a coherent net flow through the channel core, accompanied by localized recirculation near the wall during the power-stroke phase of the metachronal wave. The latter flow organization is favorable for both efficient transport across the channel and for enhanced mixing within the ciliary layer (Ding et al. 2014).

These results demonstrate the robustness and flexibility of the model formulation and spectral algorithm proposed here for computing flows in multi-layered ciliated channels across a range of ciliary kinematics. In the following, we focus on the first model of ciliary kinematics, the translating rod model, and analyze it in depth both analytically and numerically.

**Figure 5.**
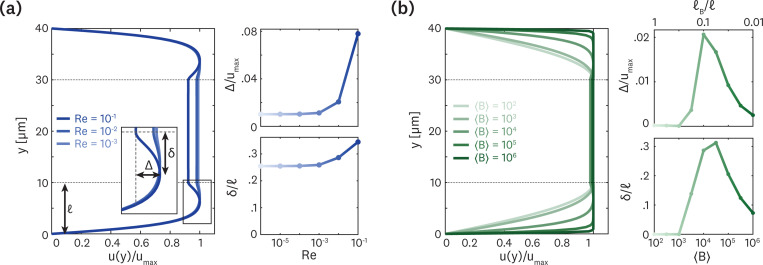
Quantifying inertial effects. Lag magnitude is denoted Δ and lag penetration depth δ. (a) Period-averaged flow profile, normalized lag magnitude, and normalized penetration depth at fixed $\langle B\rangle =10^{4}$ and varied Re. Note, u(y) profiles for Re < 10^−3^ coincide with the profile for Re = 10^−3^. (b) Period-averaged flow profile, normalized lag magnitude, and normalized penetration depth at fixed Re = 0.01 and varied ⟨B⟩. The lag magnitude and penetration depth are maximized when $\ell_{B}/\ell ∼0.1$

To conclude this section, we note that the period-averaged flow profiles from the translating-rod model at zero applied pressure exhibit a lag between the ciliary layers and the free layer (figure 4a). We quantify this effect by defining the lag magnitude Δ as the difference between the maximum velocity in the ciliary layer and the velocity at the channel center, and the penetration depth δ as the distance from the fluid-porous media interface at which the velocity decreases by 1% from its maximum (figure 5).

To characterize the origin of this lag, we perform simulations in which we vary the Reynolds number Re and mean Brinkman coefficient ⟨B⟩ independently. We find that both Δ and δ increase monotonically with Re over the range relevant to ciliary flows, indicating that the lag arises from finite unsteady and inertial effects. In contrast, their dependence on ⟨B⟩ is non-monotonic: the lag is negligible when the Brinkman screening length $\ell_{B}$ is comparable to the ciliary layer thickness ℓ, increases to a maximum at intermediate values satisfying $\ell_{B}/\ell ∼0.1$, and decreases again as $\ell_{B}$ becomes small. This behavior reflects the competition between viscous screening within the porous layer and coupling to the free flow in the inner core: weak screening suppresses the buildup of velocity within the ciliary layer, whereas strong screening dampens interactions with the free layer over short lengthscales. Together, these results show that the observed lag is governed by the interplay between inertial effects and Brinkman screening.

## 4. Mean-field model and analytical solution

In this section, we derive an analytical approximation of the inverse kinematic mapping $\chi_{c}^{-1}$ for the translating-rod model (3.1), and use its period average to obtain a reduced one-dimensional mean-field approximation of (2.1). We then solve the resulting equation analytically, which yields inexpensive predictions of the average flow profile u(y) as a function of the applied pressure, ciliary parameters and channel geometry.

We begin by performing a regular perturbation expansion in the oscillation amplitude a of the translating-rod model (3.1). Retaining terms up to $O\left(a^{2}\right)$ yields

(4.1) $$\chi_{c}^{-1}(x,t)=X(x,t)=x-a\;cos(\omega t+kx)-a^{2}k\;cos(\omega t+kx)\;sin(\omega t+kx)+O\left(a^{3}\right).$$

Substituting (4.1) into (2.8) gives, to second order,

(4.2) $$\begin{array}{rr} B(x,t) & =\frac{\left\langle B\right\rangle }{1-ak\;sin(\omega t+kx)+a^{2}k^{2}\;{cos}^{2}(\omega t+kx)},\\ u_{c}(x,t) & =-a\omega \;\sin\left(\omega t+kx\right)+a^{2}\omega k\;\cos^{2}\left(\omega t+kx\right), \end{array}$$

where $u_{c}=v_{c}\cdot e_{x}$ is the streamwise component of the ciliary velocity $v_{c}$. Averaging over one beat period yields the mean Brinkman coefficient $\bar{B}:=\langle B\rangle$ and mean ciliary speed ${\bar{U}}_{c}$, opposite to the direction of metachronal wave propagation,

(4.3) $$\bar{B}:=\left\langle B\right\rangle ,\;{\bar{U}}_{c}:=\left\langle u_{c}\right\rangle =\frac{1}{2}a^{2}\omega k.$$

We find that metachronal waves introduce an $O\left(a^{2}\right)$ drift velocity. In the absence of metachronal coordination (k=0), ${\bar{U}}_{c}$ vanishes identically.

We now derive a mean-field model for the steady state, period-averaged streamwise flow profile u(y). Exploiting the traveling-wave structure of both the forcing and the steady-state response, we replace the time-dependent Brinkman coefficient B and ciliary velocity $v_{c}$ in (2.1) by their temporal (equivalently, spatial) averages $\bar{B}=\langle B\rangle$ and ${\bar{U}}_{c}e_{x}$. By virtue of averaging, flow unsteadiness and variations in x are eliminated. Additionally, since by channel symmetry, cross-stream flows do not contribute to the net streamwise transport, we neglect the y-component of the flow field. The Navier–Stokes–Brinkman equations in (2.1) thus reduce to a steady, one dimensional Stokes–Brinkman boundary-value problem with layered structure,

(4.4) $$\left\{\begin{array}{ll} u^{''}-\bar{B}\left(u-{\bar{U}}_{c}\right)-\Delta P=0, & 0\leq y\leq \ell ,\\ u^{''}-\Delta P=0, & \ell \leq y\leq H-\ell ,\\ u^{''}-\bar{B}\left(u-{\bar{U}}_{c}\right)-\Delta P=0. & H-\ell \leq y\leq H, \end{array}\right.$$

We impose no-slip at the channel walls and continuity of velocity and shear at the interfaces at ℓ and (H-ℓ),

(4.5) $$\begin{array}{c} u(0)=u(H)=0,\;u\left(\ell^{-}\right)=u\left(\ell^{+}\right),\;u^{'}\left(\ell^{-}\right)=u^{'}\left(\ell^{+}\right)\\ {\left.u\right|}_{(H-\ell {)}^{-}}={\left.u\right|}_{(H-\ell {)}^{+}},\;{\left.u^{'}\right|}_{(H-\ell {)}^{-}}={\left.u^{'}\right|}_{(H-\ell {)}^{+}}. \end{array}$$

Solving (4.4) in each layer, enforcing the boundary conditions (4.5), and rewriting in terms of the Brinkman screening length $\ell_{B}$ yields

(4.6) $$u(y)=\left\{\begin{array}{ll} \left({\bar{U}}_{c}-\ell_{B}^{2}\Delta P\right)\left[1-cosh\frac{y}{\ell_{B}}\right]+\beta \;sinh\frac{y}{\ell_{B}}, & 0\leq y\leq \ell ,\\ \frac{1}{2}(\Delta P)\;y^{2}-\frac{1}{2}(H\Delta P)y+D, & \ell \leq y\leq H-\ell ,\\ \left({\bar{U}}_{c}-\ell_{B}^{2}\Delta P\right)\left[1-cosh\frac{H-y}{\ell_{B}}\right]+\beta \;sinh\frac{H-y}{\ell_{B}}, & H-\ell \leq y\leq H, \end{array}\right.$$

with constants

(4.7) $$\begin{array}{l} \beta =\left({\bar{U}}_{c}-\ell_{B}^{2}\Delta P\right)\tanh\frac{\ell }{\ell_{B}}-\frac{1}{2}\ell_{B}\left(H-2\ell \right)\Delta P\;sech\frac{\ell }{\ell_{B}},\\ D=\frac{1}{2}\ell (H-\ell )\Delta P+\left({\bar{U}}_{c}-\ell_{B}^{2}\Delta P\right)\left(1-sech\frac{\ell }{\ell_{B}}\right)-\frac{1}{2}\ell_{B}(H-2\ell )\Delta P\;tanh\frac{\ell }{\ell_{B}}. \end{array}$$

The solution u(y) consists of two symmetric, cilia-driven Brinkman layers adjacent to the walls and a central Stokes core. The hyperbolic structure in the outer layers reflects momentum screening over the Brinkman screening length $\ell_{B}$, while the core exhibits, in addition to the contribution from the ciliary layers, the expected parabolic profile of pressure-driven Stokes flow. We now examine the behavior of this solution in relevant asymptotic limits.

In the absence of the porous layers, for $\bar{B}=0$, (4.4) collapses to a reverse Poiseuille flow over the entire channel width, where flow is only driven by the externally-applied adverse pressure gradient ΔP (appendix C). Similarly, in the limit where the activity is zero, that is, ${\bar{U}}_{c}=0$, (4.6) and (4.7) reduce to the solution for flow through a 2D channel with homogeneous, static porous layers lining the top and bottom walls, reminiscent of the analytical solutions derived by Kuznetsov (1996); Vafai & Kim (1990). Here too, flow is only driven by the externally applied pressure gradient ΔP, and the velocity profile consists of a reverse Poiseuille-like flow profile in the center of the channel, superimposed on the screened flow within the ciliary layers.

Next, we examine the limit of strong Brinkman drag where $\bar{B}$ is large and $\ell_{B}\ll \ell$, with ${\bar{U}}_{c}$ nonzero. Here, (4.6) remains unchanged, but $tanh\left(\ell /\ell_{B}\right)\to 1$ and $sech\left(\ell /\ell_{B}\right)\to 0$, such that the constants in (4.7) become

(4.8) $$\beta ={\bar{U}}_{c}-\ell_{B}^{2}\Delta P,\;D={\bar{U}}_{c}+\frac{1}{2}\left[\ell (H-\ell )+-2\ell_{B}^{2}-\ell_{B}(H-2\ell )\right]\Delta P.$$

In this regime, pressure-driven effects are strongly attenuated within the porous layers due to momentum screening over the short length scale $\ell_{B}$. As a result, even when the applied pressure gradient is sufficient to reverse the flow in the free core, the flow within the ciliary layers remains largely unaffected; reversing it requires an adverse pressure of order $\ell_{B}^{-2}$.

In the same strong Brinkman-drag regime, setting ΔP=0 yields,

(4.9) $$u(y)=\left\{\begin{array}{ll} {\bar{U}}_{c}\left[1-cosh\frac{y}{\ell_{B}}\right]+{\bar{U}}_{c}\;sinh\frac{y}{\ell_{B}}, & 0\leq y\leq \ell ,\\ {\bar{U}}_{c}, & \ell \leq y\leq H-\ell ,\\ {\bar{U}}_{c}\left[1-cosh\frac{H-y}{\ell_{B}}\right]+{\bar{U}}_{c}\;sinh\frac{H-y}{\ell_{B}}, & H-\ell \leq y\leq H. \end{array}\right.$$

so that both the peak velocity in the active layers and the velocity in the core attain the ciliary speed ${\bar{U}}_{c}$. The resulting profile is plug-like, with a uniform core flow entrained at speed ${\bar{U}}_{c}$ and thin boundary layers of thickness $O\left(\ell_{B}\right)$ near the walls. This behavior is consistent with the flow profile shown in figure 4a.

## 5. Parametric analysis of ciliary transport

In this section, we examine how the morphology of a ciliated channel governs fluid transport as measured by the flow rate through the channel and the pressure generated by the ciliary layers. Because the flow rate and pressure cannot both be controlled independently, we impose an adverse pressure gradient ΔP as a way of measuring the pressure generation, while measuring the resulting flow rate Q. We begin by fixing the channel morphology and characterizing the pumping response as a function of ΔP, thereby establishing baseline performance metrics. We then vary the mean ciliary fraction ⟨Φ⟩, which controls the total amount of ciliary material in a channel of fixed geometry, and quantify its impact on pumping across the full range of ΔP. Finally, we vary the ciliary confinement ratio c, which sets the geometric extent of the ciliary layers, and examine the resulting trade-offs between throughput and pressure generation. This progression isolates the respective roles of ciliary packing and channel geometry in determining transport performance.

To quantify transport performance, we define three complementary metrics. The no-load flow rate $Q_{0}={\left.Q\right|}_{\Delta P=0}$ measures the throughput in the absence of an imposed pressure gradient, while the stall pressure $\Delta P_{s}={\left.\Delta P\right|}_{Q=0}$ denotes the maximum adverse pressure that can be sustained before the flow ceases; for $\Delta P>\Delta P_{s}$, the flow reverses and pumping fails. We also define the pumping efficiency $\eta =P_{\text{out}}/P_{\text{in}}$ as the ratio of useful hydraulic power to the power injected by the ciliary layers, where the output power $P_{\text{out}}=Q(\Delta P)\Delta P$ is the work performed against the imposed pressure, and the input power $P_{\text{in}}=\int_{0}^{L}\int_{0}^{H}Bv_{c}\cdot v_{c}\;dx\;dy$ is the rate of mechanical work exerted by the active porous medium. Together, $Q_{0}$, $\Delta P_{s}$, and η quantify the ability of the system to generate flow, sustain pressure, and convert input power into useful transport.

In the mean-field model introduced in section 4, these quantities ($Q_{0}$, $\Delta P_{s}$, and η) can be evaluated explicitly. In particular, the input power admits a closed-form expression based on the prescribed kinematics, yielding $P_{\text{in}}=2\ell L\bar{B}\left(a^{2}\omega^{2}/2+3{\left(a^{2}\omega k\right)}^{2}/8\right)$, which provides a direct link between ciliary motion and energetic cost.

**Figure 6.**
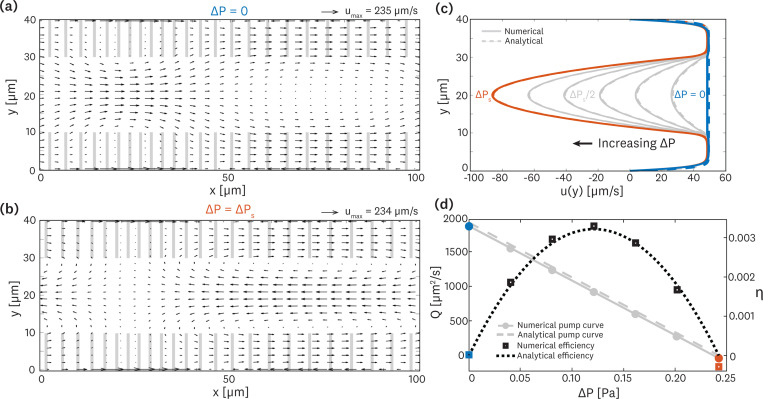
Flow field and pumping performance for fixed channel geometry and ciliary fraction. (a) Flow field at zero applied pressure ΔP=0, i.e. the no-load flow. (b) Flow field at stall, $\Delta P=\Delta P_{s}$ with zero net flow Q=0. (c) Period averaged flow profiles with corresponding mean-field analytical predictions (dashed). Blue curves indicate ΔP=0, orange curves indicate $\Delta P=\Delta P_{s}$, and gray curves indicate intermediate values of ΔP. (d) Pump curve (linear) and efficiency curve (quadratic). Colored markers indicate no-load (blue) and stall (orange) corresponding with the curves in (c). Here, ⟨Φ⟩=0.2, $\bar{B}=3.4\times 10^{4}$, and H=40μm, corresponding to c=0.5. For all simulation results plotted here and thereafter, a=5μm, ω=10Hz, d=0.2μm, ℓ=10μm, L=100μm, and Re=0.01.

We first examine the flow generated by prescribed ciliary activity for a fixed morphology, varying only the imposed pressure ΔP. In figure 6, representative steady-state velocity fields at no load (ΔP=0) and at stall $\left(\Delta P=\Delta P_{s}\right)$ are shown in panels (a,b), with the corresponding streamwise period-averaged velocity profiles u(y) in panel (c).

The resulting pump curve Q versus ΔP is shown in figure 6d. The pump curve is well described by the linear relation

(5.1) $$Q\left(\Delta P\right)=Q_{0}\left(1-\frac{\Delta P}{\Delta P_{s}}\right),$$

which interpolates between the no-load and stall limits. Next, to evaluate the pumping efficiency η, we substitute (5.1) into the expression for the output power. We get that $P_{\text{out}}=Q_{0}\Delta P\left(1-\Delta P/\Delta P_{s}\right)$ is a quadratic function of ΔP that vanishes at both no load and stall and attains its maximum at $\Delta P=\Delta P_{s}/2$, corresponding to $Q=Q_{0}/2$. Because the input power $P_{\text{in}}$ is independent of ΔP, the efficiency η is maximized at the same operating point, with $\eta_{\text{max}}=Q_{0}\Delta P_{s}/\left(4P_{\text{in}}\right)$. This quadratic dependence of η on ΔP, with a maximum at $\Delta P_{s}/2$, is confirmed by the numerical results in figure 6d.

Results from the linearized analytical solution (4.6)–(4.7) are superimposed in figure 6c,d. For the value of $\bar{B}$ considered here, the mean-field model accurately captures both the velocity profiles and the pump curve, as evidenced by the agreement between analytical (dashed) and numerical (solid) results.

The linear dependence of the flow rate Q on ΔP, together with the corresponding quadratic dependence of the η on ΔP, provides a baseline against which the effects of ciliary packing and confinement are assessed next.

**Figure 7.**
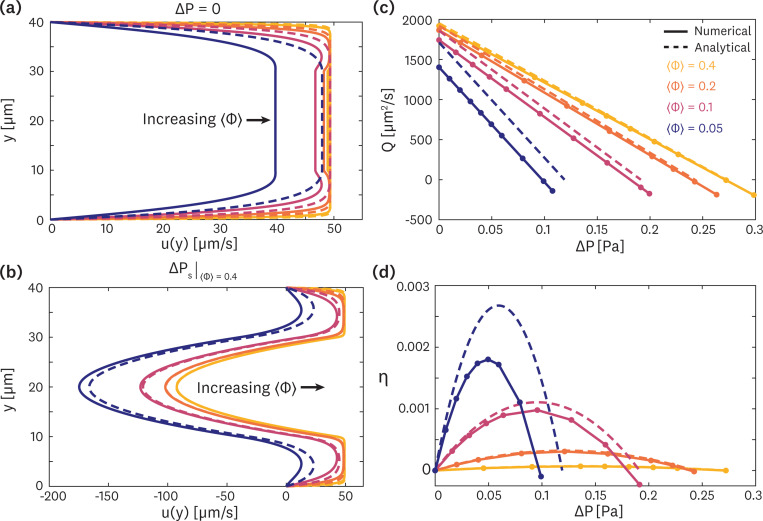
Flow profiles and pumping performance for varied ciliary fraction and fixed confinement ratio. Analytical predictions are indicated with dashed lines, color indicates the ciliary fraction ⟨Φ⟩, with warmer colors indicating more material and higher $\bar{B}$. (a) Period averaged flow profiles at ΔP=0. (b) Period averaged flow profiles at the stall pressure of the largest ⟨Φ⟩ plotted. (c) Pump curves corresponding with the set of profiles plotted in (a,b). More ciliary material increases both the no-load flow rate and stall pressure. (d) Efficiency curves corresponding with the pump curves in (c). Here H=40μm and c=0.5. ⟨Φ⟩=[0.05,0.1,0.2,0.4] corresponds with nondimensional Brinkman coefficient $\bar{B}=\left[1.8\times 10^{3},7.6\times 10^{3},3.4\times 10^{4},1.8\times 10^{5}\right]$.

In figure 7, we vary the mean ciliary fraction ⟨Φ⟩, which controls the total amount of ciliary material, while keeping the ciliary confinement ratio c fixed. Increasing ⟨Φ⟩ enhances the Brinkman drag within the ciliary layers, thereby reducing the screening length and localizing momentum transfer near the walls. This effect is evident in the period-averaged velocity profiles (figure 7a,b), where larger ⟨Φ⟩ produces steeper velocity gradients within the ciliary layers and stronger shear at the interface with the free fluid. As a consequence, both the no-load flow rate and the stall pressure increase with ⟨Φ⟩ (figure 7c), reflecting the enhanced ability of the ciliary layer to both drive flow and sustain adverse pressure gradients. However, this increase in transport capacity comes at the expense of efficiency: the pumping efficiency decreases with increasing ⟨Φ⟩ (figure 7d), indicating that in this parameter range, denser ciliary packing incurs a higher energetic cost per unit transported fluid.

**Figure 8.**
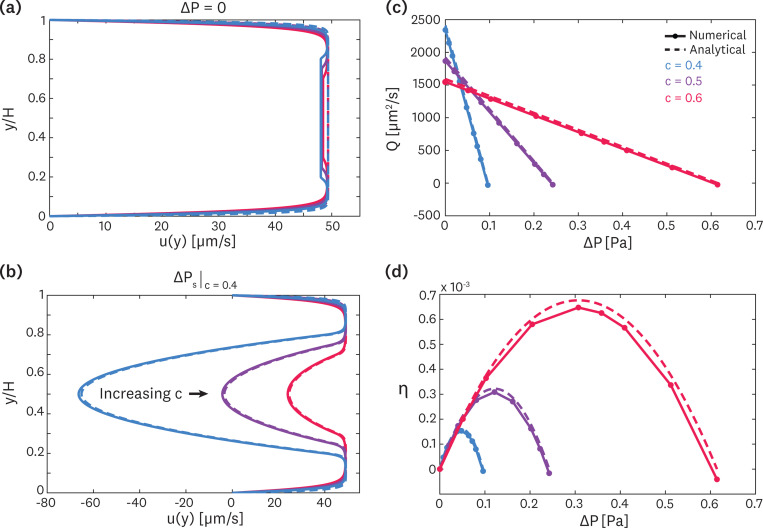
Flow profiles and pumping performance for fixed ciliary fraction and varied confinement ratio. (a) No load flow profiles plotted on normalized y axes. (b) Flow profiles at the stall pressure of the smallest 2ℓ/H. Increasing the ciliated fraction allows the pumps to sustain larger adverse pressure before flow reversal. (c) Pump curves corresponding with the profiles in (a,b). Larger ciliated fraction, and thus smaller channel height H, decreases the no load flow rate and increases the stall pressure. (d) Efficiency curves corresponding with the pump curves in (c). c=[0.4,0.5,0.6] corresponds with H=[50,40,33.33]. For all panels, ciliary material is fixed at ⟨Φ⟩=0.2 corresponding with reference Brinkman coefficient $\bar{B}=3.4\times 10^{4}$.

Next, we fix the total amount of ciliary material and vary the ciliary confinement ratio c by holding ℓ fixed and varying the channel height H (figure 8). This variation introduces a trade-off between bulk transport and the ability to sustain flow against adverse pressure. At no load (figure 8a), the flow generated within the ciliary layers entrains fluid in the channel interior even as H increases, leading to an increase in the no-load flow rate as c decreases. In contrast, under an imposed adverse pressure gradient (figure 8b), channels with smaller c exhibit pronounced backflow in the center region, resulting in a reduced stall pressure. These trends are reflected in the pump curves shown in figure 8c. Because the total ciliary material is fixed, the input power remains constant across this set of simulations. The increase in stall pressure $\Delta P_{s}$ with increasing c outweighs the corresponding decrease in no-load flow rate $Q_{0}$, so that the overall pumping efficiency η increases with confinement.

**Figure 9.**
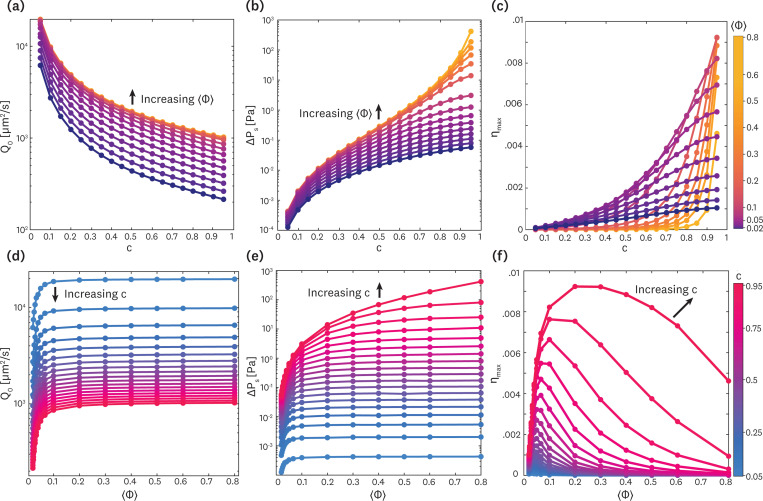
Numerical results for varied confinement ratio (a-c) and ciliary fraction (d-f). (a) No-load flow rate versus confinement ratio. Increased fraction increases the no load flow rate at every confinement ratio. (b) Stall pressure versus confinement ratio. Increased ciliary fraction increases the stall pressure at every confinement ratio. (c) Maximum pumping efficiency versus confinement ratio. The amount of ciliary material non-monotonically influences the maximum pumping efficiency. (d) No-load flow rate versus ciliary fraction ⟨Φ⟩. Increased confinement ratio decreases the no-load flow rate because the channel size shrinks. (e) Stall pressure versus ciliary fraction. Increased confinement ratio increases the stall pressure. (f) Maximum pumping efficiency versus ciliary fraction. For each confinement ratio, there exists and optimal amount of ciliary material for which the maximum pumping efficiency is maximized. ⟨Φ⟩=[0.02-0.8] corresponds with interciliary spacings $b=\left[10-0.25\right]\mu m$, and $\bar{B}=\left[1.8\times 10^{2}-2.2\times 10^{6}\right]$. c=[0.05-0.95] corresponds with H=[400-21]μm.

In figure 9, we summarize the numerically computed transport performance over the full range of ciliary confinement ratio c and mean ciliary fraction ⟨Φ⟩. In the top row (figure 9a–c), the no-load flow rate $Q_{0}$, stall pressure $\Delta P_{s}$, and maximum pumping efficiency $\eta_{max}$ are shown as functions of c for different values of ⟨Φ⟩, while the bottom row (figure 9d-f) presents the same quantities as functions of ⟨Φ⟩ for different values of c. Each curve in the bottom row therefore corresponds to a vertical slice of the results shown in the top row.

Consistent with the trends identified in figures 7 and 8, the no-load flow rate (figure 9a,d) decreases with increasing c and increases with increasing ciliary fraction ⟨Φ⟩, with the latter dependence saturating for ⟨Φ⟩≈0.1-0.2. In contrast, the stall pressure increases monotonically with both c and ⟨Φ⟩ (figure 9b,e), exhibiting a particularly strong sensitivity to confinement: increasing c from 0.05 to 0.95 leads to an increase in stall pressure spanning several orders of magnitude. Saturation with respect to ⟨Φ⟩ is observed at moderate confinement but is delayed as c increases.

The maximum pumping efficiency (figure 9c,f) exhibits a non-monotonic dependence on ⟨Φ⟩. For each c value, there exists an optimal ciliary fraction that maximizes efficiency, resulting in a well-defined peak. As c increases, both the optimal ⟨Φ⟩ and the corresponding maximum efficiency shift to larger values. These results demonstrate that transport performance is governed by a coupled dependence on ciliary fraction and geometric confinement.

**Figure 10.**
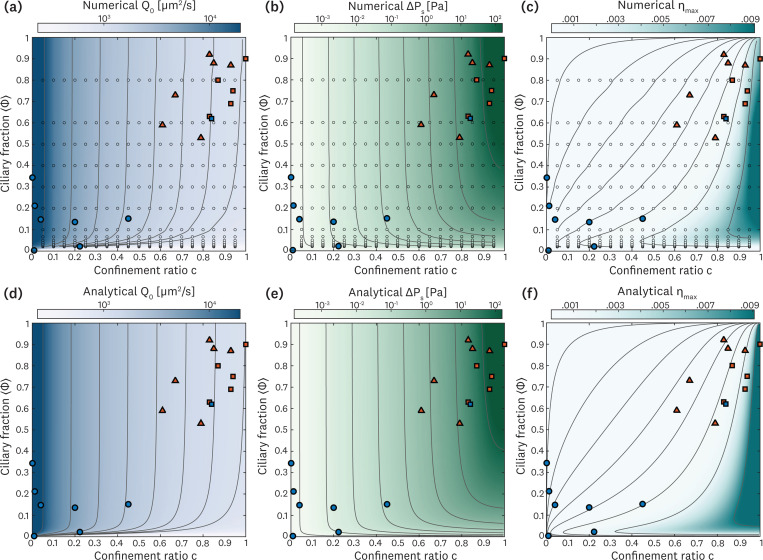
Model results overlaid with biological data. Numerical no-load flow rate (a), stall pressure (b), and maximum pumping efficiency (c). Analytical no-load flow rate (d), stall pressure (e), and maximum pumping efficiency (f). Contour lines in indicate equal elevation and are spaced evenly in percentiles of the data. Colored markers correspond with the biological data in figure 1 and table 4. Blue markers indicate ciliary carpets and orange markers ciliary flames. Circles represent ducts used for bulk transport, triangles for filtration, and squares for ducts with unknown functions. Small circles in (a-c) indicate numerical simulations, in between the markers the colormaps are interpolated, outside of the simulation region, the colormap is extrapolated.

**Figure 12.**
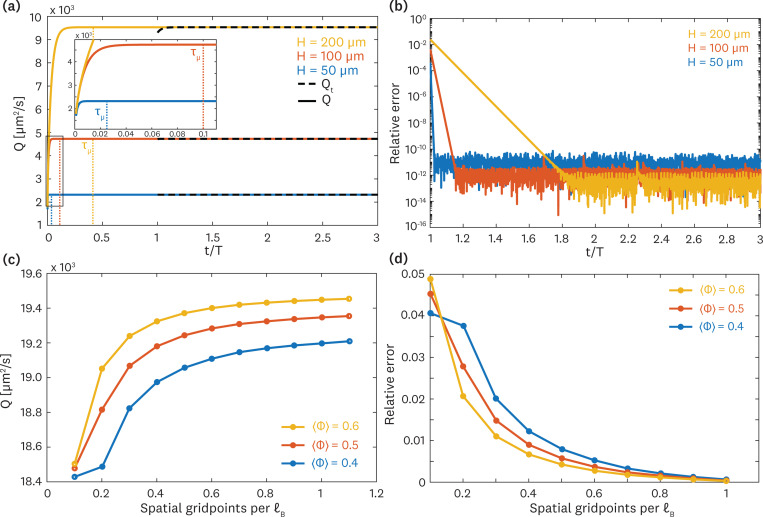
Convergence of the numerical solver. (a,b) Convergence to a periodic steady state. (c,d) Spatial resolution study. (a) Instantaneous flow rate obtained by spatial averaging over one metachronal wavelength (solid lines) is compared with the period-averaged flow rate computed from temporal averaging at a fixed location (dashed lines). Results are shown for L=100μm and H=50,100,200μm with $\Phi_{\text{ref}}=0.2$, using one grid point per Brinkman screening length $\ell_{B}\left(M_{x}=N_{y}=92,\;184,\;370\right)$. The solution approaches a periodic steady state on the viscous diffusion timescale $\tau_{\mu }$, indicated by vertical dotted lines. (b) Relative difference between the two flow-rate measures. The comparison begins at t/T=1, once temporal averaging over a full beat period becomes available. The subsequent decay reflects convergence to the periodic steady state on the timescale $\tau_{\mu }$. (c) Steady-state flow rate Q as a function of spatial resolution, measured in grid points per $\ell_{B}$, for $\Phi_{ref}=0.4,\;0.5,\;0.6$. (d) Relative error in Q, computed with respect to the highest-resolution case (1.2 points per $\ell_{B}$). A minimum resolution of $M_{x}=N_{y}=32$ is imposed, which sets the lowest-resolution point.

**Figure 13.**
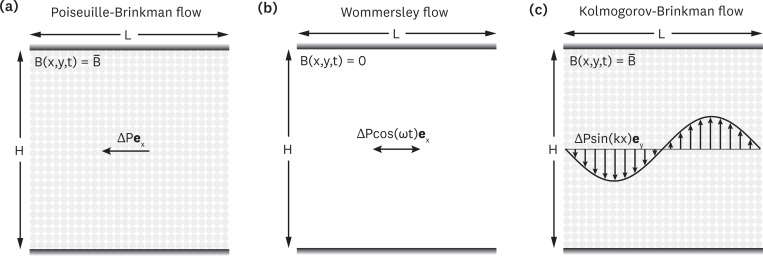
Validation tests. (a) Poiseuille–Brinkman flow driven by a constant body force $\Delta P\;e_{x}$ in a homogeneous porous medium with Brinkman coefficient $\bar{B}$. (b) Womersley flow driven by an oscillatory body force $\Delta P\;cos(\omega t)\;e_{x}$ in the absence of Brinkman drag $\left(\bar{B}=0\right)$. (c) Kolmogorov-Brinkman flow driven by a spatial body force $\Delta P\;sin(kx)\;e_{y}$ with homogeneous Brinkman coefficient $\bar{B}$.

**Figure 14.**
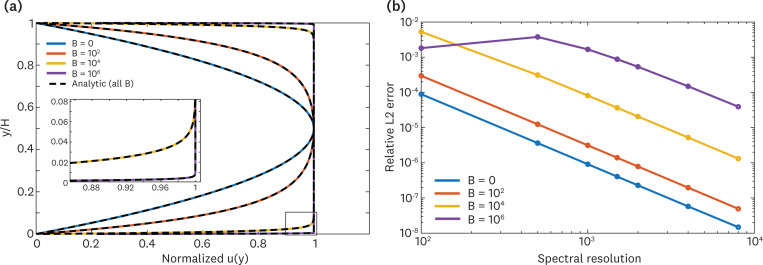
Validation using Poiseuille–Brinkman flow. (a) Normalized velocity profiles for pressure-driven flow with homogeneous Brinkman coefficient $B=0-10^{6}$ at the highest tested resolution $M_{x}=N_{y}=8000$. Numerical solutions (solid lines) agree with analytical solutions (dashed lines). Each profile is normalized by its maximum; increasing B sharpens the near-wall gradients (inset). (b) Relative $L^{2}$ error versus spectral resolution $M_{x}=N_{y}$. Second-order spatial convergence is observed once the Brinkman screening length is resolved. Parameters: H=L=100μm, Re=0.01, ΔP=0.1.

**Figure 15.**
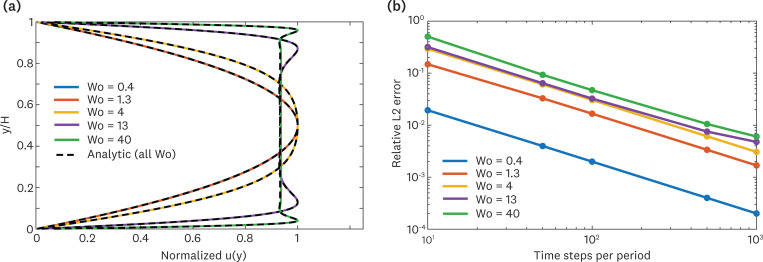
Validation using oscillatory Womersley flow. (a) Normalized maximum amplitude velocity profiles for Womersley numbers $W_{o}=0.4-40$ at high temporal (1000 time steps per period) and spatial ($M_{x}=\left.N_{y}=800\right)$ resolution. Numerical (solid) and analytical (dashed) solutions are indistinguishable; the profiles for $W_{o}=0.4$ and 1.3 overlap. (b) Relative $L^{2}$ error, averaged over one period at steady state, versus time steps per period. First-order temporal convergence is observed. Parameters: ω=[0.1,1,10,100,1000]Hz, H=L=1mm, Re=1, B=0, ΔP=0.01.

**Figure 16.**
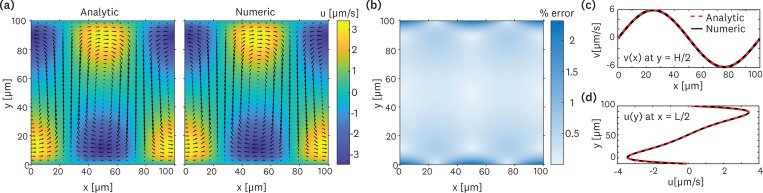
Validation using wall-bounded Kolmogorov-Brinkman flow. (a) Analytical flow field (left) and numerical flow field (right). Color bar indicates the magnitude of the horizontal component of the flow u. (b) Percent error between the analytical and numerical flow fields, as measured by $100\times |u_{\text{num}}-u_{\text{ana}}|/\left(max\left(\left|u_{\text{ana}}\right|\right)\right)$. (c) Vertical component of the flow v(x) at y=H/2. (d) Horizontal component of the flow u(y) at x=L/2. Red dashed line indicates the analytical curve, solid black the numerical curve. Here, $M_{x}=480$ and $N_{y}=960$. The wavenumber k is set so that there’s a single waveform per L. Parameter values are L=H=100μm, Re=1, $\bar{B}=100$, and $\Delta P=10^{-3}$.

Figure 10 summarizes the numerical and analytical results in the (c,⟨Φ⟩) parameter space. The top (analytical) and bottom (numerical) rows report the no-load flow rate, stall pressure, and maximum pumping efficiency. These maps reveal a fundamental trade-off between bulk transport and pressure generation. The no-load flow rate is maximized at small confinement c and intermediate ciliary fraction ⟨Φ⟩≈0.1, beyond which further increases in ⟨Φ⟩ yield only marginal gains. In contrast, the stall pressure increases monotonically with both c and ⟨Φ⟩ and is maximized at large confinement and high ciliary fraction. The maximum pumping efficiency is optimized at intermediate ⟨Φ⟩≈0.1-0.2 and large c. These optimal regions do not coincide, indicating that a ciliated channel cannot simultaneously maximize throughput, pressure generation, and efficiency.

The analytical model accurately captures the no-load flow rate, stall pressure, and maximum efficiency across most of parameter space, with deviations exceeding 10% confined to the regime of weak ciliary packing, ⟨Φ⟩≲0.1, where the assumptions underlying the mean-field approximation break down.

Placing these results in a biological context, the measurements of (c,⟨Φ⟩) reported in figure 1c cluster in distinct regions of parameter space. Ciliary carpets lie near the regime that maximizes the no-load flow rate with the minimal amount of ciliary material, while ciliary flames occupy the regime of high stall pressure. This alignment is consistent with their respective physiological roles: carpets are specialized for high-throughput transport in relatively unconstrained environments, whereas flames operate in narrow, resistive ducts where pressure generation is critical for filtration and pumping. Notably, neither class of systems lies near the region of maximal hydrodynamic efficiency, suggesting that energetic optimality is not the primary design constraint in these systems.

Overall, these results demonstrate that ciliary transport is governed by a fundamental geometric and material trade-off: low confinement and moderate ciliary fraction favor throughput, whereas strong confinement and dense ciliary packing favor pressure generation. This trade-off provides a unifying physical framework for interpreting the diversity of ciliated transport systems observed in biology.

These results also raise a natural question: why not combine the advantages of both transport regimes by constructing a large-diameter ciliary flame with high ciliary confinement and high ciliary fraction? Such a configuration could, in principle, exploit the enhanced throughput associated with large ducts while simultaneously maintaining the strong pressure generation characteristic of densely packed ciliary layers. A ciliary flame, however, requires sufficiently long and densely packed cilia to span and effectively fill the duct cross-section (figure 1b). Because cilia length is biologically constrained, flame-like morphologies can at best be sustained in ducts whose diameter is comparable to the cilia length, namely H≤ℓ. This explains why ciliary flames in nature tend to occur in narrow ducts lined with long, densely packed cilia and suggests that the trade-off between throughput and pressure generation identified here arises, at least in part, from biological constraints on achievable cilia length and packing density – constraints that could potentially be relaxed in engineered active transport systems.

## 6. Discussion

In this work, we developed a unified theoretical framework for cilia-driven transport in confined channels by modeling ciliary layers as an active porous medium governed by a Navier–Stokes–Brinkman formulation. This approach bridges the gap between classical envelope models, which treat the ciliary layer as an impermeable boundary (Blake 1971, 1972), and filament-resolved simulations that explicitly capture individual cilia dynamics and hydrodynamic interactions (Ding et al. 2014; Guo et al. 2014; Nawroth et al. 2017; Man et al. 2020; Man & Kanso 2020). By introducing a coarse-graining procedure that, for prescribed ciliary kinematics, links the mean ciliary fraction to an effective Brinkman drag, we identified a minimal set of parameters—namely, the ciliary confinement ratio c and the mean ciliary fraction ⟨Φ⟩—that govern transport. Within this framework, we showed that ciliary pumping is characterized by a linear pressure–flow relation, a quadratic efficiency curve, and a fundamental trade-off between throughput and pressure generation, consistent with mean-field descriptions of active flows (Ling et al. 2024).

Exploring this parameter space—the space of ciliary confinement ratio c and mean ciliary fraction ⟨Φ⟩—revealed that low confinement and moderate ciliary fraction favor high-throughput transport, whereas strong confinement and dense ciliary packing enhance the ability to sustain adverse pressure gradients, consistent with predictions from a simpler model in Ling et al. (2024). These regimes align with biological observations: ciliary carpets, which operate in relatively wide lumens and exhibit coordinated metachronal waves, cluster in the high-throughput regime (Kanale et al. 2022; Ling et al. 2024), while ciliary flames, which function in narrow, resistive ducts, lie in the pressure-generating regime (Ling et al. 2024). This correspondence supports the view that the diversity of ciliary architectures reflects underlying physical constraints imposed by flow and geometry, rather than a single universal optimal design.

While our results align with available biological data, several aspects of ciliated transport remain to be incorporated. The present model reduces geometric and material complexity to two effective parameters, enabling a clear connection between morphology and function, but does not capture the full structural richness of biological systems (Ramirez-San Juan et al. 2020; Ling et al. 2024). Many ciliated ducts exhibit spatially varying diameters, curvature, and patchy ciliation, with coverage fractions that vary along the organ length (Ramirez-San Juan et al. 2020). In addition, some systems transport suspended particles or interact with complex, non-Newtonian fluids such as mucus, and all involve mechanical feedback between the cilia and the surrounding fluid (Ding et al. 2014; Nawroth et al. 2017; Roth et al. 2025). These effects are neglected here but are expected to play an important role in shaping transport in specific physiological contexts. A key advantage of the active porous media framework is that such features can be incorporated systematically, allowing increasing biological realism while maintaining computational tractability.

Beyond ciliated systems, the physical mechanisms identified here may extend more broadly to biological transport processes driven by coordinated active motion. In particular, recent work on plant–fungal symbiosis has shown that traveling-wave-like strategies can regulate nutrient exchange across interconnected networks (Oyarte Galvez et al. 2025). Despite the different biological context, these systems share key features with ciliary transport, including spatially distributed forcing, wave-like coordination, and flows in geometric confinement. Similar principles have also been identified in intracellular flows driven by active filament assemblies, where geometry and activity jointly regulate large-scale transport (Stein & Shelley 2019; Dutta et al. 2024; Chakrabarti et al. 2024; Jain et al. 2025). This suggests that the trade-off between throughput and resistance identified here may represent a general organizing principle governing transport in active biological media.

These findings also have implications for the design of bio-inspired microfluidic pumps (Gauger et al. 2009; Vilfan et al. 2010; den Toonder & Onck 2013; ul Islam et al. 2022; Hanasoge et al. 2018; Liu et al. 2026b). In particular, the identified trade-off between throughput and pressure generation provides a clear guideline for tuning device performance: low confinement and moderate actuation density favor high flow rates, whereas strong confinement and dense actuation enhance pressure generation. This suggests that different operating regimes can be targeted by adjusting geometric confinement and the effective actuation of the driving elements, without requiring detailed control at the level of individual actuators.

Taken together, our results provide a minimal physical framework for understanding how geometry and active forcing jointly determine transport in confined systems (Ling et al. 2024). By identifying a small set of governing parameters and the trade-offs they impose, this work offers a unifying perspective on cilia-driven transport and highlights broader connections to other forms of biologically mediated flow, from epithelial ciliary carpets to intracellular streaming and symbiotic transport networks (Kanale et al. 2022; Dutta et al.2024; Oyarte Galvez et al. 2025).

## Figures and Tables

**Figure 11.**
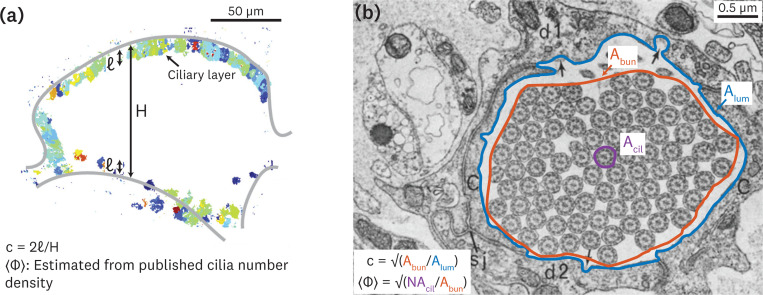
Representative images of ciliary lumen from published literature. (a) Cross section of the larval zebrafish brain ventricle Olstad et al. (2019). Colored regions indicate regions of ciliary activity. We measured the confinement ratio as the average of ℓ and H taken from multiple locations throughout this cross section. The ciliary fraction was estimated from the published cilia number density Olstad et al. (2019). (b) Cross section of protonephridial ciliary flame in planaria McKanna (1968). We manually traced the outlines of the lumen and the ciliary bundle to obtain their areas. The confinement ratio is computed as the square root of the bundle area divided by the lumen area. The ciliary fraction is the square root of the average area of one cilia multiplied by the number of cilia, divided by the bundle area.

**Table 1.** Ciliated channel parameters: Representative dimensional values compiled from the literature (Gilpin et al. 2020; Ling et al. 2024; Roth et al. 2025), together with their nondimensional counterparts obtained using the characteristic channel length L, ciliary speed $U=\omega \ell =100\;\mu m\;s^{-1}$, and fluid viscosity $\mu =10^{-3}\;N\;s\;m^{-2}$. Average values of the ciliary fraction and Brinkman coefficient, denoted by ⟨⋅⟩ and based on biological observations, are used in the reference configuration.

Quantity	Symbol	Dimensional value		Non-dimensional value
Channel length	L	100	μm	1
Channel height	H	20–400	μm	0.2–4
Ciliary layer thickness	ℓ	10	μm	
Ciliary confinement ratio	c=2ℓ/H	―		0–1
Ciliary fraction	Φ(x,y,t)	―		⟨Φ⟩=0-1
Brinkman coefficient	B(x,y,t)	$\langle B\rangle =10^{7}-10^{11}$	$N\cdot s\cdot m^{-4}$	$\bar{B}=\langle B\rangle =10^{2}-10^{6}$
Adverse pressure	ΔP	0–400	$N\cdot m^{-2}$	0–10^5^

**Table 2.** Scaling from nondimensional to dimensional values. Lengths are scaled by the channel length L, velocities by U=ωℓ, time by L/U, pressure by μU/L, and the Brinkman coefficient by $\mu /L^{2}$. Throughout, dimensional values are obtained using L=100μm, $U=100\;\mu m\;s^{-1}$, and $\mu =10^{-3}N\;s\;m^{-2}$.

Quantity	Non-dimensional	Scaling to dimensional
Streamwise Eulerian coordinate	x	Lx
Wall-normal Eulerian coordinate	y	Ly
Time	t	(L/U)t
Fluid velocity vector field	u(x,y,t)	Uu
Fluid pressure field	p(x,y,t)	(μU/L)p
Ciliary velocity vector field	$v_{c}(x,y,t)$	$Uv_{c}$
Brinkman drag coefficient	B(x,y,t)	$\left(\mu /L^{2}\right)B$
Adverse pressure gradient	ΔP	(μU/L)ΔP

**Table 3.** Cilia beat kinematics: parameters defining the three planar kinematic models introduced in section 2.2 and figures 2 and 3. In all cases, the phase lag θ(X,t)=ωt+kX, with k=2π/L, correspond to a single metachronal wavelength over the channel length L.

Model	Mapping $\chi_{c}$	Parameters
Translation	(X+acosθ,Y)	a, ω, k
Rotation	$(X+Ysin\;\alpha ,\;Y\;cos\;\alpha ),\;\alpha =\alpha_{0}\;sin\;\theta$	$\alpha_{0}$, ω, k
Kinematics from data	$\left(X+x_{c}(Y,\theta ),\;y_{c}(Y,\theta )\right)$	$A_{0x}$, $A_{0y}$, $A_{x}$, $B_{x}$, $A_{y}$, $B_{y}$, ω, k

**Table 4.** Biological ciliated channels: mean ciliary fraction and confinement ratio estimated from published data.

Ciliary carpets						

Species	Common name	Organ	Coverage	⟨Φ⟩	c	References

*Homo sapiens sapiens*	Human	Airway	0.86	0.34	0.001	Roth et al. (2025)
*Rattus norvegicus*	Rat	Brain ventricle	–	0.003	0.009	Kazanis & Ffrench-Constant (2012) Haemmerle et al. (2015)
*Rattus norvegicus*	Rat	Airway	0.53	0.21	0.01	Roth et al. (2025)
*Mus musculus*	Mouse	Airway	0.37	0.15	0.04	Ramirez-San Juan et al. (2020) Boucher et al. (2024); Li et al. (2021)
*Homo sapiens sapiens*	Human	Fallopian tube	0.34	0.14	0.20	Raidt et al. (2015); Varga et al. (2018)
*Danio rerio*	Larval zebrafish	Brain ventricle	–	0.02	0.22	Olstad et al. (2019)
*Saccocirrus papillocercus*	Ringed worm	Foregut	0.38	0.15	0.45	Purschke & Tzetlin (1996)
*Dicyema acuticephalum*	Dicyema	Urn cavity	–	0.62	0.84	Furuya et al. (1997)
Ciliary flames						

Species	Common name	Organ	Coverage	⟨Φ⟩	c	References

*Meiopriapulus fijiensis*	Penis worm	Protonephridia	–	0.59	0.61	Storch et al. (1989)
*Thalassema thalassemum*	Spoon worm	Head kidney	–	0.73	0.67	Kato et al. (2011)
*Artioposthia sp.*	Land planaria	Protonephridia	–	0.53	0.79	Rohde & Watson (1992)
*Habrotrocha rosa*	Rotifer	Protonephridia	–	0.92	0.83	Schramm (1978)
*Lepidochitona corrugata*	Mollusk	Metanephridia	–	0.63	0.83	Baeumler et al. (2012)
*Urnatella gracilis*	Goblet worm	Protonephridia	–	0.88	0.85	Kümmel (1962)
*Danio rerio*	Larval zebrafish	Metanephridia	–	0.80	0.87	Ott et al. (2016)
*Dugesia tigrina*	Planarian flatworm	Protonephridia	–	0.87	0.93	McKanna (1968)
*Aeolosoma bengalense*	Polychaete worm	Metanephridia	–	0.69	0.93	Bunke (1994)
*Geotrypetes seraphini*	Caecilian	Mesonephros	–	0.75	0.94	Møbjerg et al. (2004)
*Oikopleura dioica*	Larvacean	Ciliated funnel	–	0.90	1.00	Holmberg (1982); Ling et al. (2024)

## Appendix A. Estimating ciliary fraction and confinement ratio from data

We estimate from biological data two independent geometric quantities that enter the mathematical model: the mean ciliary fraction ⟨Φ⟩ and the ciliary confinement ratio c. In our two-dimensional formulation, Φ(x,y,t) denotes the local ciliary fraction, defined as the area fraction occupied by ciliary material within the ciliated layer, and ⟨Φ⟩ denotes its spatial average. The ciliary confinement ratio is a global measure of the fraction of the channel occupied by the ciliary layers, defined as c=2ℓ/H, where ℓ is the ciliary layer thickness and H is the channel diameter.

**Algorithm B.1.** Precompute Eulerian Brinkman field $B\left(x,y,t^{n}\right)$ and ciliary velocity field $v_{c}\left(x,y,t^{n}\right)$.

1:	**Input:** $\bar{B}$, $\chi_{c}(X,Y,t)$, current time $t^{n}$, Eulerian grid, time step Δt
2:	Evaluate forward map: $\chi_{c}^{n}(X,Y)=\chi_{c}\left(X,Y,t^{n}\right)$
3:	Compute deformation gradient: $F^{n}=\nabla_{X}\chi_{c}^{n}$
4:	Compute Jacobian: $J^{n}=det\;F^{n}$
5:	Compute ciliary velocity: $v_{c}^{n}(X,Y)=\partial_{t}\chi_{c}\left(X,Y,t^{n}\right)$
6:	Push forward Brinkman coefficient: $B^{n}(X,Y)=\bar{B}/J^{n}(X,Y)$
7:	**for** each Eulerian grid point (x,y) **do**
8:	Invert mapping: $(X,Y)=\chi_{c}^{-1}\left(x,y,t^{n}\right)$
9:	Set $v_{c}^{n}(x,y)=v_{c}^{n}(X,Y)$
10:	Set $B^{n}(x,y)=B^{n}(X,Y)$
11:	**end for**
12:	Store $v_{c}^{n}(x,y)$ and $B^{n}(x,y)$

The procedure used to estimate ⟨Φ⟩ depends on the available biological data. When the cilia number density $\rho_{c}$ (number of cilia per unit area) is reported, we estimate the characteristic center-to-center spacing as $b=\rho_{c}^{-1/2}$ and define the corresponding mean ciliary fraction as $\langle \Phi \rangle ∼d\;\rho_{c}^{1/2}$. When $\rho_{c}$ is not available, we estimate it indirectly by combining a representative per-cell fraction (400 cilia per $100\;\mu m^{2}$ (Nanjundappa et al. 2019)) with the coverage fraction of ciliated cells, obtained from image analysis. The resulting $\rho_{c}$ is then used to compute ⟨Φ⟩ as above.

For ciliary flames, the geometry differs from that of carpets, and cross-sectional images provide direct access to the relevant area fractions. We therefore estimate both c and ⟨Φ⟩ from images taken perpendicular to the channel axis. From these images, we trace the channel boundary, the extent of the ciliary bundle, and the individual cilia cross-sections. An example of this analysis is provided in figure 11b. The ciliary layer thickness 2ℓ is defined as the square root of the area occupied by the ciliary bundle and the lumen characteristic height H is the square root of the lumen area. The confinement ratio c is then defined as c=2ℓ/H, as above. The ciliary fraction ⟨Φ⟩ is defined as the square root of the area occupied by cilia within the ciliary bundle, divided by the area of the bundle. This square root converts cross-sectional area fractions into effective linear fractions consistent with the reduced two-dimensional model (figure 2). Refer to the supplementary data for notes on the analysis of each ciliated organ.

## Appendix B. Numerical flow solver

To numerically solve the nondimensional Navier–Stokes–Brinkman equation (2.1) subject to the boundary conditions (2.2), we developed a custom pseudo-spectral solver in MATLAB. To this end, we expand the velocity field in Fourier modes in the periodic streamwise coordinate x∈[0, 1] and sine modes in the wall-normal coordinate scaled to the unit interval, y/h∈[0,1], where h=H/L,

(B 1)
$$u(x,y,t)=\sum_{m=-M_{x}/2}^{M_{x}/2-1}\sum_{q=1}^{N_{y}}{\overset{ˆ}{u}}_{mq}(t)e^{i2\pi mx}\;sin\left(\frac{\pi qy}{h}\right).$$

Here, $M_{x}$ denotes the number of Fourier modes in the x direction and $N_{y}$ the number of sine modes in the y direction. We consider even $M_{x}$ and adopt the notation $m=-M_{x}/2,\ldots ,M_{x}/2-1$ accordingly. This choice of basis automatically enforces periodicity in x and no-slip conditions at the walls y=0 and y=h.

To rewrite (2.1) on this y-scaled domain, we use the dimensionless gradient and Laplacian operators

$$\nabla =\left(\partial_{x},\frac{1}{h}\partial_{y}\right),\;\nabla^{2}=\partial_{xx}+\frac{1}{h^{2}}\partial_{yy}.$$

We solve the resulting equations numerically by employing a first-order implicit–explicit time-stepping scheme together with a projection method to enforce incompressibility. Specifically, we evaluate the nonlinear advection term u⋅∇u explicitly in physical space and the stiff linear terms (diffusion and Brinkman drag) implicitly in spectral space (Kutz 2013), thereby avoiding the severe time-step restriction $\Delta t≲Re/B_{\text{max}}$, where $B_{\text{max}}$ denotes the largest value of the dimensionless Brinkman field.

**Algorithm B.2.** Implicit-explicit pseudo-spectral solver for the Navier–Stokes–Brinkman equations.

**Require:** Predefined adverse pressure gradient ΔP, channel aspect ratio h=H/L	
**Require:** Initial velocity $u^{0}$, time step Δt	
1:	**for** $n=0,1,\ldots ,N_{t}-1$ **do**
2:	Represent $u^{n}(x,y)$ using Fourier modes in x and sine modes in y
3:	Compute Eulerian ciliary fields $B\left(x,y,t^{n+1}\right.$ and $v_{c}\left(x,y,t^{n+1}\right)$ according to Algorithm B.1
4:	Compute nonlinear term (B 2) in physical space
5:	Assemble linear system (B 3) in spectral space
6:	Solve (B 3) for intermediate velocity $u^{*}$ using a matrix-free Krylov method and $\bar{B}$ preconditioner
7:	Solve the pressure Poisson equation (B 5) in spectral space
8:	Project onto divergence-free velocity field (B 4)
9:	Store $u^{n}(x,y)$
10:	**end for**
11:	Compute and store streamwise x-averaged flow profile u(y) and flow rate Q(t) according to (3.5)

To this end, we write the nonlinear term in skew-symmetric form,

(B 2)
$$N\left(u^{n}\right)=\frac{1}{2}\left[\left(u^{n}\cdot \nabla \right)u^{n}+\nabla \cdot \left(u^{n}⊗u^{n}\right)\right],$$

and evaluate it in physical space. We substitute back into the Navier-Stokes-Brinkman (2.1), ignoring for now the incompressibility constraint, to obtain a set of linear equations for the intermediate velocity $u^{*}$,

(B 3)
$$\left(I+\frac{\Delta t}{Re}\left(-\nabla^{2}+B^{n+1}I\right)\right)u^{*}=u^{n}-\Delta t\;N\left(u^{n}\right)+\frac{\Delta t}{Re}\left(\Delta Pe_{x}+B^{n+1}v_{c}^{n+1}\right)$$

where I denotes the identity operator and I denotes the 2×2 identity tensor. We solve this variable-coefficient linear system using a matrix-free Krylov method (MATLAB pcg) with a diagonal preconditioner constructed from the constant reference field $\bar{B}$.

To enforce incompressibility, we project $u^{*}$ onto a divergence-free field by seeking a pressure increment ϕ such that

(B 4)
$$u^{n+1}=u^{*}-\frac{\Delta t}{Re}\nabla \phi ,\;\nabla \cdot u^{n+1}=0.$$

Taking the divergence of (B4) yields the Poisson equation

(B 5)
$$\nabla^{2}\phi =\frac{Re}{\Delta t}\nabla \cdot u^{*}.$$

We solve equation (B5) in spectral space using Fourier modes in x and cosine modes in y, enforcing homogeneous Neumann boundary conditions at the walls. The divergence-free velocity $u^{n+1}$ is then obtained from (B4). Algorithm B.2 presents pseudocode for the numerical method, and full details of the validation and convergence of the scheme are provided in appendix C and figures 12–16.

We assess temporal convergence and determine the time required to reach a periodic steady state by comparing two measures of the flow rate. At each time step, we compute the spatially averaged flow rate Q over one metachronal wavelength (defined in equation (3.5)), and the period averaged flux at a given instant t

(B 6)
$$Q_{t}=\frac{1}{T}\int_{t}^{t+T}\;\int_{0}^{H}u\left(x_{0},y,t\right)\;dy\;dt.$$

Agreement between Q and $Q_{t}$ indicates that a periodic steady state has been reached. We find that this occurs on the viscous diffusion timescale $\tau_{\mu }=\rho H^{2}/\mu$. In practice, we simulate for $1.25\;\tau_{\mu }$ and require that the relative change in Q between successive time steps be below $5\times 10^{-4}$.

To determine spatial resolution, we vary the number of grid points relative to the Brinkman screening length $\ell_{B}$. We find that resolving $\ell_{B}$ with at least 0.4 grid points yields relative errors below $O\left(10^{-2}\right)$ in the flow rate. All simulations therefore use a minimum of 0.4 grid points per $\ell_{B}$, with a resolution floor of 256 × 256 modes.

Temporal convergence is assessed by varying the time step over a range of resolutions. We observe first-order convergence, with relative errors in the flow rate below $O\left(10^{-3}\right)$ at sufficiently small time steps. Based on this, we use $\Delta t=2.5\times 10^{-4}$, corresponding to 400 time steps per ciliary beat period, for all simulations in the main text.

## Appendix C. Validation of numerical flow solver

To validate the pseudo-spectral Navier-Stokes–Brinkman solver and the projection scheme, we compare numerical solutions against analytical results for three canonical flows: (i) pressure-driven Poiseuille–Brinkman flow, (ii) oscillatory Womersley flow, and (iii) wall-bounded Kolmogorov flow with homogeneous Brinkman drag illustrated in figure 13. In each case, the numerical solution is advanced in time until a steady or periodic steady state is reached and is then compared to the corresponding closed-form solution.

We first consider the classical Poiseuille flow corresponding to flow between two no-slip walls in the absence of a porous medium. The nondimensional Navier–Stokes equations (2.1) reduces to the well-known one-dimensional equation, subject to no-slip boundary conditions at the channel walls,

(C 1)
$$\frac{d^{2}u(y)}{dy^{2}}=-\Delta P,\;u(0)=u(H)=0.$$

Integrating twice yields the classical parabolic profile,

(C 2)
$$u(y)=\frac{\Delta P}{2}y(H-y).$$

We next consider the Poiseuille–Brinkman flow, by including a homogeneous, static Brinkman medium by setting $v_{c}=0$ and $B=\bar{B}$. The steady governing equation, again with no-slip boundary conditions, becomes

(C 3)
$$\frac{d^{2}u(y)}{dy^{2}}-\bar{B}u(y)=-\Delta P,\;u(0)=u(H)=0,$$

for which the analytical solution is given by

(C 4)
$$u(y)=\frac{\Delta P}{\bar{B}}\left[1-\frac{cosh\left(\sqrt{\bar{B}}\left(y-\frac{H}{2}\right)\right)}{cosh\left(\frac{\sqrt{\bar{B}}H}{2}\right)}\right].$$

Testing these cases in the numerical solver corresponds to setting $\bar{B}=0$ and $v_{c}=0$ for the Poiseuille flow, and setting $B=\bar{B}$ everywhere with $v_{c}=0$ for the Poiseuille–Brinkman flow, while imposing a constant adverse pressure gradient ΔP. The solution is advanced in time to steady state and compared with the analytical profiles (C2) and (C4). As shown in figure 14, excellent agreement is obtained, confirming second-order spatial convergence and validating the numerical treatment of the viscous and Brinkman terms.

To validate the temporal integration scheme and the implicit treatment of viscous diffusion, we consider oscillatory pressure-driven flow between two no-slip walls (Womersley flow). In the absence of Brinkman drag and ciliary forcing, the equations reduce to a one-dimensional unsteady Stokes problem with no-slip boundary conditions,

(C 5)
$$\frac{\partial u}{\partial t}=\frac{1}{Re}\frac{\partial^{2}u}{\partial y^{2}}+\Delta P\;cos(\omega t),\;u(0,t)=u(H,t)=0,$$

where u(y,t) is the streamwise velocity and ΔPcos(ωt) is a spatially uniform, oscillatory body force representing an imposed oscillatory pressure gradient. We seek a time-harmonic analytical solution of the form $u(y,t)=R\left\{\overset{ˆ}{u}(y)e^{i\omega t}\right\}$, where R{⋅} denotes the real part of a complex quantity. Substitution into (C5) yields a second-order boundary-value problem for the complex amplitude $\overset{ˆ}{u}(y)$. Solving for $\overset{ˆ}{u}(y)$, multiplying by $e^{i\omega t}$, and taking the real part we arrive at

(C 6)
$$u(y,t)=R\left\{\frac{\Delta P}{i\omega }\left[1-\frac{cosh\left(\sqrt{i\omega Re}\left(y-\frac{H}{2}\right)\right)}{cosh\left(\frac{\sqrt{i\omega Re}\;H}{2}\right)}\right]e^{i\omega t}\right\}.$$

Figure 15a shows normalized analytical and numerical velocity profiles at peak amplitude for the forcing frequencies considered. The Womersley number, $Wo=(H/2)\sqrt{\rho \omega /\mu }$, measures the ratio of oscillatory to viscous timescales. Excellent agreement is obtained at sufficient temporal resolution. Varying the time step confirms first-order temporal convergence (figure 15b).

Lastly, to validate the full spectral solver and projection method, we consider steady, wall-bounded Kolmogorov flow with homogeneous Brinkman drag. The governing equations are

(C 7)
$$-\nabla p+\nabla^{2}u-\bar{B}u+\Delta P\;sin(kx)e_{y}=0,\;\nabla \cdot u=0,$$

subject to periodic boundary conditions in x and no-slip conditions at y=0 and y=H. The body force $\Delta P\;sin(kx)e_{y}$ varies sinusoidally in x and drives a fully two-dimensional flow.

We seek a single Fourier-mode solution of the form

(C 8)
u(x,y)=U(y)cos(kx),v(x,y)=V(y)sin(kx),p(x,y)=P(y)sin(kx).

Substitution into (C7) and incompressibility yield

(C 9)
$$V^{'}(y)=k\;U(y),$$

which allows elimination of V and P, leading to the fourth-order ordinary differential equation

(C 10)
$$U^{\prime \prime \prime \prime }-\left(2k^{2}+\bar{B}\right)U^{''}+\left(k^{4}+\bar{B}k^{2}\right)U=0.$$

Let $\lambda =\sqrt{k^{2}+\bar{B}}$. The general solution of (C10) is

(C 11)
$$U\left(y\right)=A\;\sinh\left(ky\right)+\beta \;\cosh\left(ky\right)+C\;\sinh\left(\lambda y\right)+D\;cosh(\lambda y).$$

The constants are determined from the no-slip conditions U(0)=U(H)=0 and the wall momentum balance

(C 12)
$$U^{'''}(y)-\left(2k^{2}+\bar{B}\right)U^{'}(y)=k\Delta P\;\text{at}\;y=0,H.$$

Solving for (y) first, then for V(y) and P(y), we get

(C 13)
$$\begin{array}{l} U\left(y\right)=A\;\sinh\left(ky\right)+\beta \;\cosh\left(ky\right)+C\;\sinh\left(\lambda y\right)+D\;cosh(\lambda y),\\ V\left(y\right)=A\left[\cosh\left(ky\right)-1\right]+\beta \;sinh(ky)+\frac{k}{\lambda }C[cosh(\lambda y)-1]+\frac{k}{\lambda }D\;sinh(\lambda y),\\ P(y)=-\frac{\bar{B}}{k}(A\;sinh(ky)+\beta \;cosh(ky)). \end{array}$$

The coefficients satisfy

(C 14)
$$A=-\frac{\Delta P}{\lambda^{2}}-\frac{k}{\lambda }C,\;\beta =-D,$$

with C and D given by

(C 15)
$$\begin{array}{r} C=\Delta P\frac{\frac{k}{\lambda }sinh(kH)(cosh(\lambda H)-cosh(kH))-(cosh(kH)-1)\left(sinh(\lambda H)-\frac{k}{\lambda }sinh(kH)\right)}{k\lambda (cosh(\lambda H)-cosh(kH){)}^{2}+\left(sinh(\lambda H)-\frac{k}{\lambda }sinh(kH)\right)\left(\lambda^{2}sinh(kH)-k\lambda sinh(\lambda H)\right)},\\ D=\Delta P\frac{(cosh(\lambda H)-cosh(kH))\left(cosh(kH)-1-\frac{k}{\lambda }sinh(kH)\right)}{k\lambda (cosh(\lambda H)-cosh(kH){)}^{2}+\left(sinh(\lambda H)-\frac{k}{\lambda }sinh(kH)\right)\left(\lambda^{2}sinh(kH)-k\lambda sinh(\lambda H)\right)}. \end{array}$$

To simulate this flow numerically, we impose the sinusoidal body force in (C7) and advance the solution in time to steady state, defined by a relative change in the maximum velocity below 10^−4^ between successive time steps. Figure 16 shows excellent agreement between numerical and analytical solutions over all parameter values tested, validating the solver for fully two-dimensional flows.

In all validation simulations, the maximum divergence remained $O\left(10^{-12}\right)$ or smaller, confirming that the projection method (appendix B) enforces incompressibility to machine precision.
